# Increasing prevalence of a fluoroquinolone resistance mutation amongst *Campylobacter jejuni* isolates from four human infectious intestinal disease studies in the United Kingdom

**DOI:** 10.1371/journal.pone.0227535

**Published:** 2020-01-30

**Authors:** Sam Haldenby, Christina Bronowski, Charlotte Nelson, John Kenny, Carmen Martinez-Rodriguez, Roy Chaudhuri, Nicola J. Williams, Ken Forbes, Norval J. Strachan, Jane Pulman, Ian N. Winstanley, Caroline E. Corless, Tom J. Humphrey, Frederick J. Bolton, Sarah J. O’Brien, Neil Hall, Christiane Hertz-Fowler, Craig Winstanley

**Affiliations:** 1 Centre for Genomic Research, University of Liverpool, Liverpool, United Kingdom; 2 Institute of Infection and Global Health, University of Liverpool, Liverpool, United Kingdom; 3 Department of Biology and Biotechnology, University of Sheffield, Sheffield, United Kingdom; 4 School of Medicine, Medical Sciences & Nutrition, Foresterhill, University of Aberdeen, Aberdeen, United Kingdom; 5 School of Biological Sciences, University of Aberdeen, Aberdeen, United Kingdom; 6 Infection and Immunity, Liverpool Clinical Laboratories, Royal Liverpool and Broadgreen University Hospitals NHS Trust, Liverpool, United Kingdom; 7 Medical Microbiology and Infectious Diseases, School of Medicine, Swansea University, Swansea, United Kingdom; 8 Department of Public Health and Policy, Institute of Population Health Sciences, University of Liverpool, Liverpool, United Kingdom; 9 NIHR Health Protection Research Unit in Gastrointestinal Infections, University of Liverpool, Liverpool, United Kingdom; 10 The Earlham Institute, Norwich Research Park, Norwich, United Kingdom; 11 School of Biological Sciences, University of East Anglia, Norwich, United Kingdom; Defense Threat Reduction Agency, UNITED STATES

## Abstract

**Background:**

*Campylobacter jejuni* is the most common bacterial cause of human infectious intestinal disease.

**Methods:**

We genome sequenced 601 human *C*. *jejuni* isolates, obtained from two large prospective studies of infectious intestinal disease (IID1 [isolates from 1993–1996; n = 293] and IID2 [isolates from 2008–2009; n = 93]), the INTEGRATE project [isolates from 2016–2017; n = 52] and the ENIGMA project [isolates from 2017; n = 163].

**Results:**

There was a significant increase in the prevalence of the T86I mutation conferring resistance to fluoroquinolone between each of the three later studies (IID2, INTEGRATE and ENIGMA) and IID1. Although the distribution of major multilocus sequence types (STs) was similar between the studies, there were changes in both the abundance of minority STs associated with the T86I mutation, and the abundance of clones within single STs associated with the T86I mutation.

**Discussion:**

Four population-based studies of community diarrhoea over a 25 year period revealed an increase over time in the prevalence of the T86I amongst isolates of *C*. *jejuni* associated with human gastrointestinal disease in the UK. Although associated with many STs, much of the increase is due to the expansion of clones associated with the resistance mutation.

## Introduction

Worldwide, the zoonotic pathogen *Campylobacter* is the most common bacterial cause of acute gastroenteritis, responsible for an estimated 500,000 infections per annum in the United Kingdom (UK) alone [1], representing an economic burden in excess of £1 billion each year [2]. Campylobacteriosis, characterised by bloody diarrhoea, cramping, abdominal pain and fever, and sometimes nausea and vomiting, is typically self-limiting and lasts for about a week. However, occasionally more serious infections can occur, either because the pathogen enters the bloodstream, or it is associated with a peripheral nervous system disorder, Guillain-Barré Syndrome. The majority of cases of campylobacteriosis are caused by *C*. *jejuni* (approximately 90% [3]).

In the UK, two large scale prospective studies of infectious intestinal disease incidence and aetiology (Infectious Intestinal Disease Studies 1 and 2 [IID1 (1993–1996) and IID2 (2008–2009), respectively]) identified *Campylobacter* as the most common bacterial pathogen among both a community cohort and cases presenting to healthcare [1, 4].

Although most people with *Campylobacter* infections recover without treatment, antibiotic therapy using macrolides or fluoroquinolones is recommended for the treatment of severe *Campylobacter* infections [5], and there are concerns about increasing resistance. The World Health Organization recently included fluoroquinolone-resistant *Campylobacter* amongst the high priority pathogens against which new antibiotics are needed [6]. Specific mutations in the *gyrA* gene, most notably the C257T mutation (T86I in the protein sequence), have been associated strongly with resistance to fluoroquinolones such as ciprofloxacin [7–11].

Multilocus sequence typing (MLST), based on analysis of sequences from several housekeeping genes, has emerged as the key molecular technique in the study of diversity amongst *Campylobacter* populations and the relationships between species within the genus [12, 13]. Using MLST, several studies have reported the prevalence of specific clones (sequence types [STs]) amongst *C*. *jejuni* isolates from diverse sources [14–17], demonstrating that whilst some MLST clonal complexes, such as the ST-21 complex, are widespread, others, such as the ST-61 complex, have a more restricted distribution. MLST has also been used to identify correlations between the T86I mutation associated with ciprofloxacin resistance and specific clonal groups of *C*. *jejuni* [18, 19]. By determining the prevalence of specific sequence types amongst isolates from potential sources, and comparing with similar data from isolates associated with infections in humans, various studies have estimated the relative contributions of particular sources to transmission to humans [16, 17, 20]. This genetic attribution approach has confirmed that the majority of human infections are associated with retail chicken meat [16, 17].

More recently, whole genome sequencing (WGS) has emerged as an affordable alternative to MLST, offering far greater resolution and opportunities to improve our understanding of both the epidemiology and the fundamental biology of bacterial pathogens [21]. Since the first *Campylobacter* genome sequence (of strain NCTC11168) was published in 2000 [22], other studies involving relatively small numbers of isolates have been conducted, revealing extensive within-species diversity [23, 24]. It has been demonstrated that WGS analysis can be used to predict antibiotic resistance in *C*. *jejuni* [25].

The aim of this study was to use whole genome sequencing to compare UK human *C*. *jejuni* isolates from collections representing different time periods [IID1 (1993–1996), IID2 (2008–2009) and two more recent studies (INTEGRATE and ENIGMA; 2016–2017)].

## Materials and methods

### *C*. *jejuni* isolates

The *C*. *jejuni* isolates used in this study were obtained from collections generated by four different UK studies: IID1[4], IID2[1], INTEGRATE[26] and ENIGMA. IID1 (1993–1996) and IID2 (2008–2009) were two large scale prospective studies of infectious intestinal disease incidence and aetiology. In IID1 and IID2, bacteria were isolated from faecal samples obtained from patients of all ages attending 70 and 88 General Practitioner Practices respectively, across the UK. Stored isolates from the IID1 and IID2 studies were obtained from Public Health England. The INTEGRATE project (http://www.integrateproject.org.uk) involved collection of faecal samples from unselected cases of acute gastroenteritis of all ages presenting to primary care across the north west of England (population 7.2 million) during the period 2016–2017. *Campylobacter* isolates were obtained using *Campylobacter* Blood Free CCDA selective agar (E & O Laboratories Ltd.) and incubated for 48 h at 42°C.

In the ENIGMA project (http://www.enigmaproject.org.uk), clinical isolates of *Campylobacter* from under five year olds were collected over the period August 2016 –October 2017 (n = 195) from 17 participating hospital diagnostic microbiology laboratories in England, all with appropriate ethical approval in place. At the receiving laboratory the cultures on charcoal swabs were recultured on to mCCDA plates, and the plates incubated at 37°C for 48 h under microaerobic conditions. The presence or absence of *Campylobacter* colonies was determined visually and confirmed by visible agglutination with Microscreen *Campylobacter* latex confirmation assay (product code M46, Microgen Bioproducts).

A total of 601 *C*. *jejuni* isolates comprising 293 from IID1, 93 from IID2, 52 from INTEGRATE and 163 from ENIGMA were included in the study.

### Extraction of DNA from *Campylobacter*

For the majority of isolates, genomic DNA was extracted using a QIAamp kit (QIAGEN), following the manufacturer’s instructions. Bacteria from single colonies were cultured on Columbia blood agar incubated at 37°C in microaerobic conditions (CampyGen, Oxoid) for 48 h, and one or a few uniform colonies were used to inoculate 10–15 ml of Mueller Hinton broth (with *Campylobacter* growth supplement, Oxoid) in a tissue culture flask with a vented lid. Following incubation of the culture for 24 h with gentle shaking under microaerobic conditions at 37°C, cells were harvested by centrifugation (3000 x g for 10 min) and washed in 1 ml phosphate-buffered saline. After further centrifugation, the pellet was used for DNA extraction. For a minority of isolates, DNA was extracted directly from cell suspensions of bacterial cultures grown on solid media, using the Wizard Genomic DNA Purification Kit (Promega) and the manufacturer’s protocol for Gram-negative bacteria.

### Library preparation for Illumina sequencing

Libraries were constructed using the TruSeq Nano DNA Sample Preparation Kit (Illumina), and 200 ng of input material. The material was sheared using a Covaris S2 ultrasonicator following the 550 bp insert size protocol. Half (100 ng) of the sheared material was cleaned using 1.6x Sample Purification beads, and half volumes of all reagents were used throughout the protocol. Samples were prepared in a 96-well plate format and size-selected using the Sample Purification beads. Following eight cycles of amplification, the libraries were purified using Sample Purification beads. Each library was quantified using a Qubit (Thermofisher) and the size distribution was assessed using the Agilent 2100 Bioanalyzer. The samples were then pooled and the final library assessed using the Agilent 2100 Bioanalyzer and subsequently subjected to quantitative PCR using the Illumina Library Quantification Kit from Kapa on a Roche Light Cycler LC480II, according to manufacturer's instructions.

### Sequencing on the Illumina platform

Isolates from both the ENIGMA and INTEGRATE studies were sequenced by paired-end sequencing (2 x 150 bp) on the Illumina HiSeq 4000 platform (v1 chemistry). IID1 and IID2 isolates were sequenced by paired-end sequencing (2 x 100 bp or 2 x 125 bp) on the Illumina 2000 or HiSeq 2500 platforms (v3 chemistry).

### Bioinformatic analysis for Illumina-derived sequence data

Sequence-adaptor trimmed paired-end reads in FASTQ format were trimmed to remove low quality bases using Sickle 1.210 (https://github.com/najoshi/sickle) with a minimum window quality score of 25. Trimmed reads were then assembled into contigs using SPAdes [27]. Samples were filtered by assembly quality, completeness and purity. Samples with an assembly size between 1.4–2.1 MB were retained. Assemblies were analysed with BUSCO [28] and excluded from further analysis if the frequency of duplicated single-copy core orthologues exceeded 5% or the estimated completeness was lower than 95%, to remove potentially mixed isolates, and incomplete assemblies, respectively. Finally, sample reads were analysed with MetaPhlAn2 [29] and samples comprising over 5% of a species other than *Campylobacter jejuni* were removed. Following this, 601 samples were carried forward for analysis along with three reference strains: *C*.*jejuni* NCTC11168, RM1221 and 81–176, all obtained from NCBI (GenBank accession numbers: HE978252.1, CP000025.1 and CP000538.1 respectively). MLST types (using the 7 loci scheme: *aspA*, *glnA*, *gltA*, *glyA*, *pgm*, *tkt*, *uncA*) were determined for each isolate by aligning known alleles (obtained from https://pubmlst.org/campylobacter/) against assemblies using Bowtie2 [30] and selecting perfect hits. In cases where no perfect hit to an allele was detected, a novel allele was recorded and submitted to pubMLST (https://pubmlst.org/campylobacter/). Similarly, novel profiles were also recorded and submitted.

The pan-genome of all remaining samples was calculated using LS-BSR [31], and a core genome was extracted and aligned using the LS-BSR tool extract_core_genome.py, based on genes with a BLAST score ratio of 0.8 or higher being classified as present in the genome. This yielded a core genome of 994 genes, 1,007,194 bp. A maximum likelihood phylogeny was reconstructed based on the core genome sequences using RAxML[32] (model: GTRGAMMA, 100 bootstraps) and visualised with the Interactive Tree of Life tool (iTOL [33]). Richness, Simpson’s diversity (reciprocal index and index of diversity) and equitability metrics were calculated using Python, based on isolate counts for each sequence type in each study.

*gyrA* mutations were detected as follows. Protein sequences were predicted in each assembly using Prodigal [34]. GyrA sequences were extracted by BLASTP [35] searches against the NCTC11168 GyrA sequence and aligned with MAFFT [36]. Variant sites relative to NCTC11168 were detected using a custom Python script. A similar approach was used to identify mutations in the *rplD* and *rplV* genes. Distance matrices based on SNP differences and gene/presence absence between strains were carried out using snp-dists (https://github.com/tseemann/snp-dists).

### Database submission

All genome sequence data generated have been deposited in the European Nucleotide Archive (ENA) site (study numbers: PRJEB7116 for the IID1 and IID2 isolates; PRJEB32069 for the ENIGMA isolates; PRJEB32068 for the INTEGRATE isolates). An additional figure, entitled “Phylogenetic tree based on core gene SNP phylogeny” is accessible via the link: https://itol.embl.de/tree/138253218159113541547474319.

## Results

### Comparison of MLST profiles between *C*. *jejuni* from IID1 and IID2

A summary of all the *C*. *jejuni* genome sequence data using the Illumina platform is shown in S1 Table. A summary of extracted MLST data from all 601 genomes sequenced is shown in Table 1 and S2 Table. The most common *C*. *jejuni* STs were amongst the most abundant in each of the four studies (ST-21, ST-45, ST-257 and ST-48). For other STs, there were examples of variations in prevalence between the studies (Table 1). Some STs (eg. ST-5136, ST-6461) were present only in the later studies (INTEGRATE and ENIGMA). In the ST-21 clonal complex, there were a number of sequence types found only or mostly in the IID1 study: ST-47 (8 isolates), ST-104 (8 isolates), ST-262 (5 isolates) and ST-520 (4 isolates) (Table 1). However, it is worth noting that using SNP phylogeny, these different STs within the ST-21 clonal complex do not cluster together (Phylogenetic tree based on core gene SNP phylogeny, see Data Availability statement). In all, 23 novel MLSTs were submitted to the pubMLST.org database (ST-7628 to ST-7633; ST-7635-ST-7642; ST-9576 to ST-9580; ST-9582; ST-9583 and ST-9585).

**Table 1:** Distribution of multilocus sequence types (STs) amongst isolates from the four studies.

ST	IID1	IID2	Integrate	Enigma	TOTAL
	(n = 292)	(n = 93)	(n = 52)	(n = 162)	(n = 599)
**21**	24 (0)	7 (1)	4 (1)	16 (7)	51 (9)
**257**	29 (0)	6 (0)	1 (0)	10 (2)	46 (2)
**45**	25 (0)	3 (0)	4 (0)	11 (0)	43 (0)
**48**	14 (1)	6 (0)	2 (0)	11 (1)	33 (2)
**53**	18 (0)	4 (0)	0	6 (0)	28 (0)
**50**	12 (1)	1 (0)	6 (2)	9 (1)	28 (4)
**61**	9 (0)	2 (0)	1 (0)	4 (0)	16 (0)
**42**	7 (0)	3 (0)	0	5 (0)	15 (0)
**22**	7 (0)	2 (0)	2 (0)	2 (0)	13 (0)
**137**	6 (0)	3 (0)	0	4 (0)	13 (0)
**51**	3 (0)	3 (1)	2 (0)	4 (1)	12 (2)
**19**	5 (1)	5 (1)	1 (0)	1 (1)	12 (3)
**5136**	0	0	1 (1)	8 (8)	9 (9)
**104**	8 (1)	0	1 (1)	0	9 (2)
**52**	5 (0)	2 (0)	0	1 (0)	8 (0)
**47**	8 (0)	0	0	0	8 (0)
**464**	0	2 (2)	1 (1)	5 (5)	8 (8)
**206**	5 (0)	1 (0)	1 (0)	1 (1)	8 (1)
**354**	1 (0)	2 (2)	2 (2)	2 (2)	7 (6)
**49**	7 (0)	0	0	0	7 (0)
**267**	3 (0)	0	0	4 (0)	7 (0)
**227**	3 (2)	0	0	4 (0)	7 (2)
**6461**	0	0	0	6 (6)	6 (6)
**572**	0	4 (4)	2 (2)	0	6 (6)
**475**	4 (0)	1 (0)	0	1 (0)	6 (0)
**44**	4 (0)	0	2 (2)	0	6 (2)
**433**	5 (2)	1 (0)	0	0	6 (2)
**324**	6 (0)	0	0	0	6 (0)
**583**	3 (0)	1 (0)	0	1 (0)	5 (0)
**5**	0	1 (0)	2 (2)	2 (2)	5 (4)
**400**	0	2 (0)	1 (1)	2 (2)	5 (3)
**262**	5 (0)	0	0	0	5 (0)
**883**	0	1 (1)	0	3 (2)	4 (3)
**658**	1 (1)	0	2 (0)	1 (0)	4 (1)
**520**	4 (0)	0	0	0	4 (0)
**403**	0	0	0	4 (1)	4 (1)
**353**	4 (0)	0	0	0	4 (0)
**2254**	0	0	1 (0)	3 (3)	4 (4)
**205**	4 (0)	0	0	0	4 (0)
**132**	2 (0)	0	0	2 (0)	4 (0)
**122**	0	0	0	4 (0)	4 (0)
**singletons**	51 (6)	30 (6)	13 (4)	25 (10)	119 (26)

STs are listed in descending order of abundance. The number of isolates containing the T86I mutation is indicated in parentheses. Only STs occurring at least four times are identified in the table. Where STs occurred only once in the dataset, they are referred to as singletons. For two isolates, it was not possible to assign as ST because of incomplete profiles.

### SNP phylogeny based on the *C*. *jejuni* core genome

We constructed a maximum likelihood phylogenetic tree for *C*. *jejuni* based on a 1,007,194 bp core genome, comprising 994 genes (and 125,298 variable bases) and including the four study datasets alongside three reference strains, *C*. *jejuni* NCTC11168, RM1221 and 81–176 (Phylogenetic tree based on core gene SNP phylogeny, see Data Availability statement). The tree confirms both (i) that the IID1, IID2, INTEGRATE and ENIGMA isolates are widely distributed amongst the broader population and (ii) that there are small clusters of closely-related isolates specific to individual studies (ie. containing isolates only from the ENIGMA study).

### Comparison of diversity between the studies

We isolates compared the four studies on the basis of ST distributions to determine measures of richness, Simpson’s Diversity Index and evenness. In terms of diversity, the four groups were similar, but the IID1 isolate group was more uneven than the others (S3 Table).

### Variations in prevalence of the T86I mutation

We observed a significant increase (*p* < 0.001; Fisher’s exact test) in the prevalence of the T86I mutation between each of the three later studies (IID2, INTEGRATE and ENIGMA) and IID1 (Table 2). Increased prevalence was also observed when comparing either INTEGRATE or ENIGMA data with IID2 data (Table 2).

**Table 2:** Prevalence of the *gyrA* T86I mutation.

Source	Present	Absent	Comparison *p* values (Fisher’s exact)			
			IID1	IID2	INTEGRATE	ENIGMA
IID1	16	277	-	0.0002	<0.0001	<0.0001
IID2	18	75	-	-	0.0176	0.0148
INTEGRATE	20	32	-	-	-	0.6165
ENIGMA	55	108	-	-	-	-

The T86I mutation was present in 18% of *C*. *jejuni* isolates, but was associated with many different STs (Table 1; S2 Table). Of the 109 isolates carrying the T86I mutation, 56 (52%) were from nine STs (ST-5136, ST-21, ST-464, ST-6461, ST-354, ST-572, ST-2254, ST-5 and ST-50). Of these, ST-5136, ST-464, ST-6461, ST-572, and ST-2254 are examples of STs with only resistant isolates. None of these STs was present amongst the IID1 collection, and only ST-464 and ST-572 were represented in the IID2 collection.

**Fig 1:**
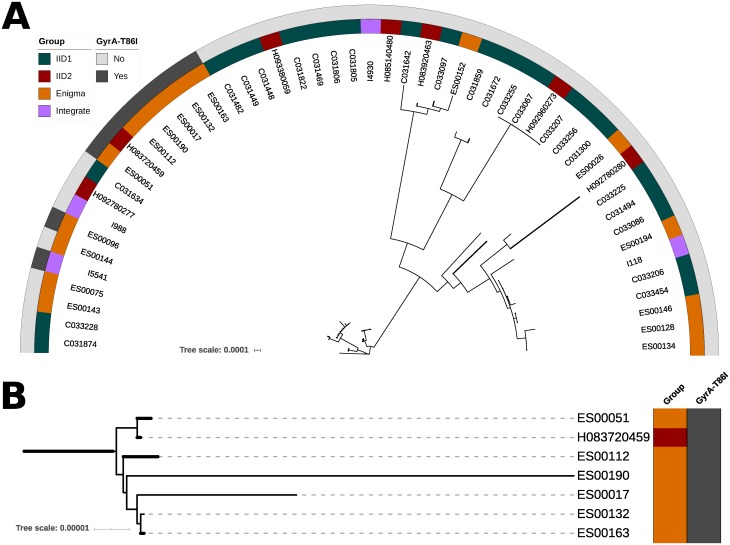
Phylogenetic tree of *C*. *jejuni* ST-21 isolates (*n* = 51) carrying the T86I mutation. (A) Inner ring identifies the study and outer ring identifies isolates carrying the GyrA T86I mutation. (B) Close up of a cluster of isolates mostly from the ENIGMA study. The tree is based on core genome SNP differences. Within this sub-set of isolates, there were 12,265 variant nucleotides.

**Fig 2:**
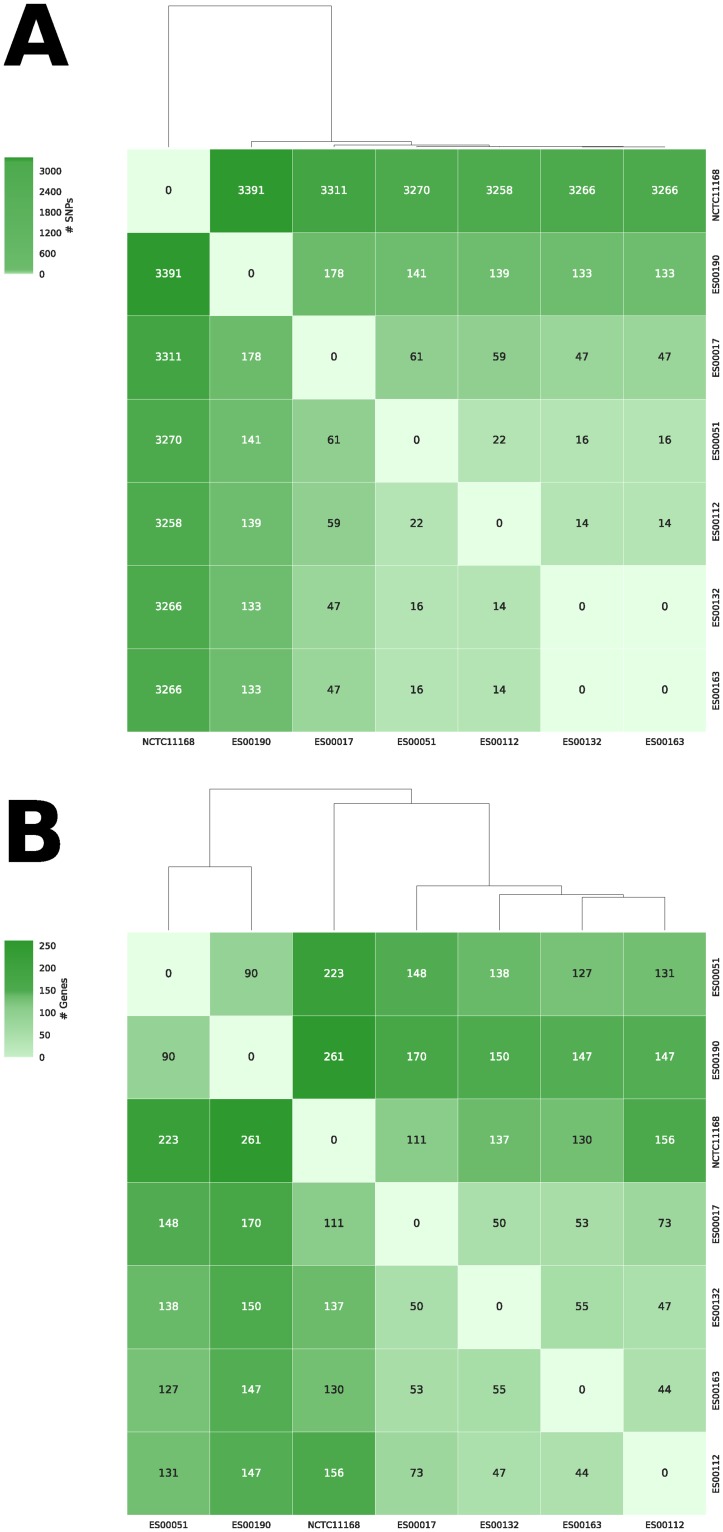
Comparative analysis of the genomes of ST-21 ENIGMA study isolates carrying the T86I mutation. The figure shows (a) the number of SNP differences and (b) the number of genes that differ in terms of presence/absence between the isolates. The reference strain NCTC11168 was included for comparison.

Though present as one of the most abundant STs in all collections, there was evidence for an increase in carriage of the T86I mutation amongst ST-21 isolates, from 0/24 in IID1to 8/20 in INTEGRATE and ENIGMA combined (*p* = 0.0007, Fisher’s exact test; Table 1). In order to investigate this further, we constructed a phylogenetic tree based on SNPs within the core genomes of only the ST-21 isolates obtained from the four studies (Fig 1). This identified a cluster including six ST-21 isolates from the ENIGMA study (ES00017, ES00132, ES00163, ES00190, ES00051 and ES00112) associated with carriage of the T86I mutation, suggesting expansion of a clone from within the ST-21 *C*. *jejuni* population. Pairwise comparisons were used to generate distance matrices based on variations in SNPs and the presence/absence of genes (Fig 2). This indicated SNP variations in the range 0–178 and gene presence / absence variations in the range 44–170. There were no core SNP differences between isolates ES00132 and ES00163, but they differed by 55 genes (Fig 2).

There was evidence for the expansion of T86I-associated clones for ST-5136, ST-6461, ST-464 and ST-572, each of which were not detected in IID1 but were present in later studies (Table 1). There were also a large number (n = 26) of singletons or rarer STs with the T86I mutation. However, it is also worth noting that there were abundant STs, such as ST-45, ST-53, ST-61, ST-42, ST-22 and ST-137 that were never associated with the T86I mutation (Table 1).

### Mutations in genes associated with macrolide resistance

In humans, the antibiotics most commonly used to treat *Campylobacter* infections are macrolides such as erythromycin. Mutations in ribosomal proteins L4 and L22 have been associated with resistance to erythromycin in *Campylobacter* [37]. We identified mutations in the genes encoding these proteins (*rplD* and *rplV*) and compared prevalence between the four groups. The only difference reaching statistical significance was an increase in prevalence of an A103V mutation in *rplV* (encoding L22), rising from 7.6% in IID1 to 19.6% in ENIGMA (*p* = 0.034). A103V mutations in L22 have been reported previously in erythromycin-resistant *Campylobacter* isolates, though the mutation can also occur in susceptible isolates [38, 39]. Mutations in the 23S rRNA gene (position 2074 or 2075) have also been implicated in resistance to erythromycin [38]. In our collection, only one isolate (H083720456) harboured such a mutation (A2075G variant).

## Discussion

Although not associated with high mortality, infections with *Campylobacter* spp. have high prevalence in the community and can cause severe infections with few treatment options. Hence, fluoroquinolone-resistant *Campylobacter* spp. have been identified amongst the high priority pathogens in the context of tackling antimicrobial resistance [6]. Using four different collections of community-based human isolates we were able to examine trends over a 25-year period and highlight differences in both the diversity of the *C*. *jejuni* population, and the carriage of a key fluoroquinolone-resistance mutation. The laboratory methods for primary diagnosis of *Campylobacter* spp. were consistent between the studies and so this should not have biased the results. Only the ENIGMA collection was restricted by age but this is compensated for by the fact that this group of strains overlapped with the INTEGRATE isolates, which were derived from cases of all ages. This suggests that the increase in prevalence of fluoroquinolone resistance witnessed in this study is real and not a result of sampling artefact. Antibiotic treatment is not advocated at any age for uncomplicated campylobacteriosis. However, we were unable to account for prescribing practice in this study. We have only limited access to data about foreign travel, but only one of the isolates carrying the T86I mutation from the INTEGRATE study was associated with foreign travel, suggesting that this was not a major source of *C*. *jejuni* containing this mutation.

The MLST database (www.mlst.net) [40] currently contains >31,000 *Campylobacter* submissions. Broadly speaking, the most common clonal complexes found amongst the *C*. *jejuni* strains in this study reflect their abundance amongst the wider *Campylobacter* population. We observed that the distributions of the major STs remained similar over time, but there were changes in the prevalence of less abundant STs. In this study we further highlight the limitations of the use of MLST clonal complexes when studying *Campylobacter* populations. We found examples of a number of clonal complexes where isolates of different STs failed to cluster together using core genome SNP phylogeny, a much higher resolution approach than MLST. Examples include clonal complexes ST-21, ST-48, ST-206 and ST-257 (Phylogenetic tree based on core gene SNP phylogeny, see Data Availability statement). In contrast, isolates of identical MLST do mostly cluster together, suggesting that analyses based on individual MLSTs may be much more robust than those based on clonal complex data. This must be borne in mind when comparing with previous studies.

Fluoroquinolone resistance amongst human and farm animal isolates of *C*. *jejuni* has been reported extensively [10, 41]. Previous studies have reported correlation between ciprofloxacin resistance (and the T86I mutation) and specific clonal groups of *C*. *jejuni* [10, 18, 19, 42, 43]. In a six year study of 3300 human *C*. *jejuni* isolates in Oxfordshire, UK, nine clonal complexes were significantly associated with ciprofloxacin sensitivity (ST- 22, ST-45, ST-48, ST-61, ST-257, ST-283, ST-403, ST-658, ST-677) and seven clonal complexes were significantly associated with ciprofloxacin resistance (ST-49, ST-206, ST-354, ST-446, ST-460, ST-464, ST-607) [44]. Likewise, in a survey of isolates from chicken meat, the ST-21 clonal complex was associated with resistance but the ST-45 clonal complex was associated with susceptibility to ciprofloxacin [10]. In agreement with these previous observations, in our study the T86I mutation was never identified in isolates from ST-45, ST-22, ST-61 but was associated with isolates from ST-354 and ST-572 (from the ST-206 clonal complex). Notably, by core genome SNP phylogeny, the ST-572 isolates do not cluster well with other members of the ST-206 clonal complex (including ST-227 and ST-206 itself). This further highlights the limitations with considering changes in terms of clonal complexes.

It has been suggested that the non-random association of MLST genotypes with resistance phenotypes is indicative of clonal expansion of resistance-associated lineages, possibly driven by the poultry industry [45]. We identified a number of STs contributing to the increased prevalence of the T86I mutation (ST-5136, ST-6461, ST-464). A previous study in Scotland, analysing isolates associated with human campylobacteriosis and poultry from 1990–2012, reported ST-5136 as a new strain emerging in the 2007–2011 period and associated with humans and chickens [46]. The isolates (>50) of ST-6461 reported in the pubMLST database were mostly from human stools in the UK and were all submitted post 2012. ST-464 was first reported in a longitudinal study of clinical samples in Oxford, UK between 2003 and 2009, increasing over that time period and associated with ciprofloxacin resistance [47].

Our data indicating increased prevalence of resistance-associated STs suggest that the main cause for the overall increased prevalence of the T86I is clonal expansion. One potential driver of this is the use of fluoroquinolones in farm animals. In the UK, fluoroquinolones have been authorised for use in poultry since 1993. There has however been much pressure on all livestock sectors to reduce antimicrobial use, and there have been specific calls to ban fluoroquinolone use completely in livestock production. However, although in the USA fluoroquinolones have been banned for use in poultry since 2005 and extra-label use is illegal, a similar rise in fluoroquinolone resistance has been observed amongst human clinical isolates, from 16.3% in 2000 to 25.3% in 2015 (https://wwwn.cdc.gov/narmsnow/). In 2012, the British Poultry Council (BPC), which represents 90% of the UK poultry industry committed to only using fluoroquinolones as last resort drugs, and to stop prophylactic use in day old chickens by 2016. Hence, fluoroquinolones have accounted for only a very small proportion of antibiotic sales (<1%) in the UK in poultry in recent years, and use has been falling. Data submitted to the Veterinary Medicines Directorate from the BPC showed a reduction of 52% in fluoroquinolone use in the poultry sector between 2014 and 2015 alone [48], with a further reduction in 2016 [49]. The most recent voluntary reporting of use data by BPC members has allowed the contribution of use within the poultry sector to be more accurately determined [50]. However, it is worth noting that some products are authorised for specific diseases, therefore veterinary surgeons must follow the legal framework of the cascade, which can conflict with good antimicrobial stewardship, where there is susceptibility to other first line drugs but they are not authorised for treatment of the disease.

If antibiotic usage is not the only driver for the expansion of lineages associated with the T86I GyrA mutation, one alternative explanation could be that the mutation confers a fitness advantage. It has been suggested that differences in the fitness costs contribute to the relatively low prevalence of macrolide resistance in *Campylobacter* spp., compared to fluoroquinolone resistance [11]. The T86I mutation hasbeen shown to reduce supercoiling GyrA activity [51] but the link between this change in the activity of the protein and the fitness of the bacteria *in vivo* is not clear. Enhanced fitness in the chicken host has been demonstrated for a fluoroquinolone resistant T86I mutant (C257T) in the absence of antibiotic selection [51, 52]. In addition, surveillance studies have shown that levels of fluoroquinolone resistance persist in poultry even after discontinued use of the antibiotics [53]. It has been demonstrated that resistance can emerge rapidly in poultry flocks [54]. It may be that once emerged, reductions in fluoroquinolone use would not be sufficient to cause a reduction in prevalence. In our study, we have observed an increased prevalence, suggesting that lineages associated with the mutation may have an advantage regardless of antibiotic use. However, it is also possible that the mutation, whilst not conferring a selective advantage, does not carry a fitness cost in a non-antibiotic environment, and hence can become fixed in the population.

## Conclusions

In conclusion, four population-based studies of community diarrhoea over a 25 year period afforded a unique opportunity to examine the prevalence of the T86I amongst isolates of *C*. *jejuni* associated with gastrointestinal disease in the UK, which has increased significantly over time despite the removal of selective pressure. Although associated with many STs, much of the increase is due to the expansion of clones associated with the resistance mutation.

## Supporting information

S1 Table A summary of the *C*. *jejuni* WGS data generated and the extracted MLST profiles (for all four studies). (XLSX)

S2 Table A summary of all isolate MLST types and the numbers of isolates carrying the T86I mutation. On the “Concise” spreadsheet, yes refers to presence of the T86I mutation and no refers to absence. The “Full” spreadsheet lists total numbers in each of the STs for each of the four studies.
(XLSX)

S3 Table Comparison of diversity between the four studies. (XLSX)
